# Expression of Toll-like receptor-9 is associated with poor progression-free survival in prostate cancer

**DOI:** 10.3892/ol.2013.1204

**Published:** 2013-02-20

**Authors:** MARJA-RIITTA VÄISÄNEN, ARJA JUKKOLA-VUORINEN, KATRI S. VUOPALA, KATRI S. SELANDER, MARKKU H. VAARALA

**Affiliations:** 1 Departments of Pathology, Oulu University Hospital, 90029 OYS;; 2 Oncology, Oulu University Hospital, 90029 OYS;; 3 Department of Pathology, Lapland Central Hospital, 96101 Rovaniemi, Finland;; 4 Department of Medicine, Division of Hematology-Oncology, University of Alabama at Birmingham, Birmingham, AL 35294-2182, USA;; 5 Department of Surgery, Oulu University Hospital, 90029 OYS, Finland

**Keywords:** prostate cancer, Toll-like receptor-9, prognosis

## Abstract

Toll-like receptor-9 (TLR9) is a member of the innate immune system and recognizes bacterial and vertebrate DNA in cells. In addition to being expressed in cells of the immune system, it is widely expressed in various types of human cancer, including prostate cancer. We have previously demonstrated that synthetic TLR9 ligands induce invasion in TLR9-expressing prostate cancer cells *in vitro*. However, the role of TLR9 in the pathophysiology of prostate cancer is unclear. The expression of TLR9 in radical prostatectomy samples (n=186) was studied using immunohistochemistry. TLR9 staining scores were compared with tumor stage, Gleason score and prostate-specific antigen (PSA) concentration prior to treatment and progression-free survival. Results revealed that 124 (66.7%) of the tumors were strongly positive, 59 (31.7%) were weakly positive and 3 (1.6%) were negative, for cytoplasmic TLR9 immunostaining in cancer cells. There was no significant association between cytoplasmic TLR9 expression and distributions of pT-class, prostatectomy sample margin status, Gleason score and preoperative PSA value. Prostate cancer-specific progression-free survival was significantly longer for patients whose tumors were graded as negative for cytoplasmic TLR9 expression, as compared with patients whose tumors were strongly immunopositive for cytoplasmic TLR9 (P=0.009). In the Cox regression analysis, high TLR9 expression was an independent marker of poor prognosis in prostate cancer. Expression of TLR9 is associated with poor progression-free survival in prostate cancer patients who were treated by radical prostatectomy with curative intent.

## Introduction

The innate immune system serves as an important first line defense against invading infections (1). Central mediators of this task are the Toll-like receptors (TLRs), which function as pattern recognition proteins that detect microbe- and host-derived molecular patterns (2). To date, 13 mammalian TLRs have been recognized, and each responds to a different ligand. For example, TLRs -4 and -5 recognize bacterial lipopolysaccharide (LPS) and flagellin, respectively, whereas the TLR9 subfamily members are nucleic acid receptors. More specifically, TLRs -7, -8 and -13 are RNA receptors, while DNA that enters the cell is recognized by TLR9 (2,3). The various receptors are expressed in different parts of a cell; TLRs -1, -2 and -4 are expressed and bind their ligands on the cell surface, while the TLR9 subfamily resides in intracellular vesicles. Binding of the cognitive ligands to the various TLRs activates transcription factors, one of the most significant being nuclear factor-κB (NF-κB). Eventually, TLR activation results in an immune response, and also in the activation of the adaptive immune system (1,2).

It is well established that in addition to being expressed in the immune system, TLR9 is widely expressed in various cancer cell lines and in clinical cancer specimens, including breast, brain, gastric, lung, esophageal, prostate and renal cancer (4–11). Previous studies have demonstrated that treatment of TLR9-expressing cancer cells with synthetic TLR9 ligands, which mimic the structure of bacterial DNA, stimulates their invasion *in vitro* through matrix metalloproteinase-13 (MMP-13) activation (6,12,13). In prostate cancer cells, native bacterial DNA had similar *in vitro* effects (12). Data concerning the regulation of TLR9 expression are starting to accumulate, although thus far, not much is known. In breast cancer cells, TLR9 expression is upregulated by sex steroids and bicalutamide (14). Testosterone has been shown to potentiate the TLR9 ligand-induced invasion of breast cancer cells, without affecting NF-κB signaling *in vitro*. However, expression of the estrogen receptor-α (ERα) inhibited these male sex hormone effects (14). Estradiol also upregulated TLR9 expression in prostate cancer cells *in vitro*(12). In malignant breast tumors, high TLR9 expression is associated with an ER^−^, and in breast cancer cells, overexpression of ERα suppresses TLR9 expression *in vitro*(14,15). Notably, steroid hormone receptors have also been shown to regulate the innate immune response in *Drosophila melanogaster*, which is suggestive of evolutionarily well-conserved regulatory pathways in this immune system (16). Hypoxia is another important regulator of TLR9 expression in cancer (17). Finally, various viral infections have been shown to downregulate TLR9 expression in normal tissues (18,19), although this has not yet been demonstrated in cancer cells.

We have previously demonstrated that the level of TLR9 expression is higher in prostate cancer than in benign hyperplasia (9). We further showed that high TLR9 expression is significantly associated with a high Gleason score (9). Previous clinical studies similarly suggest that TLR9 may contribute to the pathogenesis of various types of cancer, where high expression of TLR9 in tumors has been shown to predict decreased survival in patients with glioblastoma multiforme and esophageal cancer (7,8). A high level of TLR9 expression in prostate cancer tumor cells was also shown to be significantly associated with a higher probability of biochemical recurrence (20). On the contrary, we recently demonstrated that absent or low TLR9 expression in tumors is associated with poor prognosis in patients with renal cell carcinoma or triple-negative breast cancer, however not in ER^+^ breast cancer patients (10,17). Therefore, the impact of TLR9 expression in tumors on the prognosis of a patient appears to be dependent on the type of cancer. The aim of this study was to investigate whether expression levels of TLR9 in tumors has prognostic value in prostate cancer.

## Materials and methods

### Patient samples

Prostate specimens were obtained from an archive; these were originally collected from patients who underwent radical retropubic prostatectomy as a treatment for prostate cancer at Oulu University Hospital, Oulu, Finland, between 1996 and 2003. During this period, surgery was performed on 242 males. Following evaluation of the original diagnostic slides, six cases were excluded due to the minimal presence of carcinoma tissue. For the remaining cases, representative areas were located on the slides and the Gleason score was determined. Gleason scoring was only carried out for the prostatectomy samples, not for the diagnostic prostate biopsies. Oulu University Hospital is a tertiary referral center; patients were referred there to receive surgery after diagnosis, therefore a number of the diagnostic biopsy slides were not available. Information concerning the corresponding TNM classification and prostate-specific antigen (PSA) concentrations preceding prostatectomy were obtained from patient records. PSA concentration results were missing for a number of the patients. Time to biochemical recurrence (PSA progression leading to second line treatment), time to clinical recurrence (identification of metastases or histologically confirmed local recurrence), treatments received and the possible cause of death were also obtained from patient records. This study was approved by the Ethics Council of The Northern Ostrobothnia Hospital District.

### Immunohistochemistry

Immunohistochemical staining of the specimens was performed as previously described (9,11,17). Briefly, 4-μm sections were cut from paraffin-embedded blocks and mounted onto pre-coated slides. The sections were deparaffinized in xylene and rehydrated in descending ethanol series. To enhance immunoreactivity, the sections were incubated in a citrate buffer (pH 9.0) and boiled. Endogenous peroxidase activity was eliminated by further incubation in hydrogen peroxide and absolute methanol. A mouse monoclonal anti-human TLR9 antibody (Img-305A, clone 26C593.2; Imgenex, San Diego, CA, USA; dilution 1:200) was used to detect specific TLR9 expression. The bound antibodies were visualized using Envision Detection System (K500711; Dako, Carpinteria, Denmark A/S). Diaminobenzidine (DAB) was used as a chromogen (15). All staining was performed using the LabVision Autostainer™ (LabVision, Fremont, CA, USA).

### Evaluation of immunostaining

TLR9 immunostaining was classified as negative (0), weakly positive (+1) or strongly positive (+2). Using these criteria, the immunostained sections were evaluated by two observers (M-R.V. and M.V.) to reach a consensus.

### Statistical analysis

Statistical analysis was carried out using SPSS for Windows 15 (Chicago, IL, USA). Associations between clinicopathological variables and TLR9 immunostaining patterns were assessed using the χ^2^ test or, in the case of low expected frequencies, by the Fisher’s exact test. Progression-free survival rates were calculated using the Kaplan-Meier method, and the statistical significance between groups was analyzed using the log-rank test. Hazard ratios (HRs) were assessed using Cox univariate analysis. Progression*-*free survival rates in prostate cancer were calculated from the date of radical prostatectomy to either biochemical relapse of prostate cancer (as indicated by increase in serum PSA values) leading to second line treatments (radiation therapy or hormonal therapies), clinical progression or the last day of follow-up. Multivariate survival analysis was carried out using the Cox proportional hazards model. Two sided P-values were used. P<0.05 was considered to indicate a statistically significant result.

## Results

### TLR9 protein expression in prostate cancer

Baseline patient characteristics are provided in Table I. There were 186 prostate cancer samples available for the evaluation of TLR9 immunoreactivity. Evaluation revealed that 124 (66.7%) of the tumors were strongly positive, 59 (31.7%) were weakly positive and 3 (1.6%) were negative for cytoplasmic TLR9 immunostaining in cancer cells. For further analysis, the negative and weakly positive cases were combined and reclassified as TLR9-negative samples (n=62, 33.3%). Stromal immunoreactivity was also recorded in these specimens. The majority of the samples (n=114, 61.3%) exhibited stromal immunoreactivity to TLR9; these were classified as strongly positive (n=3, 1.6%) or weakly positive (n=111, 59.7%). The remaining 72 (38.7%) cases were negative for TLR9 immunostaining in stromal cells.

### Association of cytoplasmic TLR9 expression with clinicopathological characteristics

Twenty-five patients received neoadjuvant hormonal therapy with LHRH-analogs. These cases were excluded from the Gleason pattern analysis. Distributions of pT-class, prostatectomy sample margin status and Gleason score, and their association with cytoplasmic TLR9 expression are presented in Table II. The mean preoperative PSA values for patients with negative cytoplasmic TLR9 expression and positive cytoplasmic TLR9 expression were 15.1 ng/ml (95% CI, 8.36–21.9) and 11.2 ng/ml (95% CI, 9.21–13.2), respectively (P= 0.12). Although the results were not determined to be statistically significant, more TLR9-positive staining was observed among samples with higher Gleason scores.

### Prognostic significance of TLR9 expression in prostate cancer

**Figure 1:**
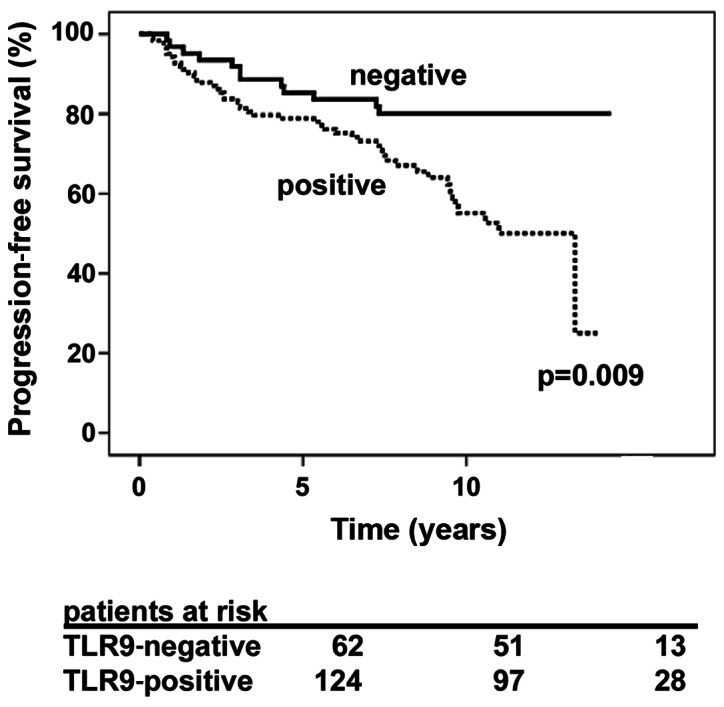
Association between cytoplasmic TLR9 expression and prostate cancer-specific progression-free survival. Patients with TLR9-negative tumors exhibited better progression-free survival rate compared with patients with tumors positive for these proteins (P= 0.009). TLR9, Toll-like receptor-9.

The prostate cancer-specific progression-free survival rate was significantly longer for patients whose tumors were graded as negative for cytoplasmic TLR9 expression, as compared with patients whose tumors were strongly immunopositive for cytoplasmic TLR9 (P=0.009; Fig. 1). The HR of patients with TLR9-expressing tumors was 2.27 (95% CI, 1.20–4.28, P=0.007). The mean prostate cancer-specific progression-free survival times for TLR9-negative and TLR9-positive tumors were 147 (95% CI, 138–161) and 116 (95% CI, 105–128) months, respectively (P=0.009). The Cox regression analysis results for age, cytoplasmic TLR9 expression and Gleason score were stratified as: Gleason score, ≤7 vs. ≥8; pT class, ≤T2c vs. T3a or T3b; and prostatectomy resection surgical margin status, negative vs. positive, as shown in Table III. Cytoplasmic TLR9 expression, Gleason score 8–10 and pT3a-pT3b, were statistically significant factors in prostate cancer-specific progression-free survival (Table III). PSA concentration was excluded from the Cox regression analysis, due to the numerous cases which were missing a preoperative PSA value. Based on the results of this study, high TLR9 expression is an independent marker of poor prognosis in prostate cancer.

## Discussion

The possible pathophysiological significance of cellular DNA receptor TLR9 in various types of cancer has attracted research interest, after studies have established that it is widely expressed in malignant tumor cells (6,9,11,12). TLR9 recognizes DNA from bacteria, viruses and the host. DNA recognition by TLR9 takes place in the endosomal-lysosomal compartment of cells. The eventual outcome of TLR9 stimulation is inflammation, characterized by increased expression of proinflammatory cytokines and other inflammatory mediators. Stimulation of TLR9 by synthetic DNA ligands or bacterial DNA also stimulates cancer cell invasion (6,12,21). Our previous studies with breast cancer cells further suggested that TLR9 expression may regulate cancer cell invasion, even in the absence of ligands (17).

Using a cohort of 186 prostate cancer samples and their associated clinical data, this study showed that high TLR9 expression in tumor cells is an independent marker of poor prognosis in prostate cancer. TLR9 staining was also detected in prostate cancer stroma, however there are no published results with regard to the prognostic role of TLR9 staining in prostate cancer stroma. Stromal staining for TLR9 appeared to be markedly less than that detected in epithelial cancer cells. Our results agree with those of Gonzáles-Reyes *et al*(20), who demonstrated that high TLR9 mRNA expression in prostate tumors is significantly associated with recurrence and higher probability of biochemical recurrence. Although the results of this study were not statistically significant, higher TLR9 expression scores were observed in tumors with higher Gleason scores. This has also been demonstrated in previous studies (9,20).

If TLR9 is significant in the pathophysiology of prostate cancer, the mechanism by which it promotes prostate cancer must be determined. There are several possibilities; firstly, TLR9 may promote the spread of prostate cancer by facilitating prostate cancer invasion. This hypothesis is supported by our previous findings, which show that knocking out TLR9 expression from cancer cells results in decreased invasion, and that *E. coli* DNA promotes prostate cancer cell invasion *in vitro*(12,17,21). Notably, testosterone promotes TLR9 ligand-induced invasion in breast cancer cells *in vitro*(14). Whether the effects are similar in prostate cancer cells requires further investigation. Secondly, TLR9 stimulation by DNA results in inflammation, which has been associated with prostate carcinogenesis (22–25). Finally, TLR9 expression appears to be upregulated by sex hormones in breast and prostate cancer cells *in vitro*(12,14). Considering the importance of sex hormones in the aggressive behavior of prostate cancer, this effect on TLR9 may also contribute to the explanation. These issues require further characterization in pre-clinical models of prostate cancer.

In conclusion, this study shows that increased expression of TLR9 is associated with poor prognosis in prostate cancer. The question that remains, however, is whether TLR9 is a driver or a passenger in prostate cancer. This should be answered via research in pre-clinical prostate cancer models, using prostate cancer cells with manipulated TLR9 expression levels.

## Figures and Tables

**Table I:** Baseline patient characteristics.

Characteristic	Value
Age (years) (n=186)	
Minimum	45
Maximum	72
Mean	62.0
PSA (ng/ml) (n=56)	
Minimum	3
Maximum	50
Mean	12.2
Androgen deprivation therapy prior to radical prostatectomy	
Yes, n (%)	25 (13.4)
No, n (%)	161 (86.6)

PSA, prostate-specific antigen.

**Table II:** Associations between cytoplasmic TLR9 expression and tumor pT-class, surgical margin status and Gleason score of the prostatectomy sample.

	Cytoplasmic TLR9 expression		
Characteristic	Negative, n(%)	Positive, n(%)	P-value
pT			
2a	13 (32)	28 (68)	0.63
2b	4 (22)	14 (78)	
2c	36 (38)	59 (62)	
3a	2 (40)	3 (60)	
3b	7 (26)	20 (74)	
Surgical margin status			
Negative	37 (33)	76 (67)	0.87
Positive	25 (34)	48 (66)	
Gleason score			
4	4 (67)	2 (33)	0.36
5	17 (40)	25 (60)	
6	20 (29)	49 (71)	
7	16 (29)	40 (71)	
8	3 (38)	5 (62)	
9	2 (40)	3 (60)	

TLR9, Toll-like receptor-9.

**Table III:** Cox multivariate progression-free survival analysis in 186 patients with prostate cancer treated using radical prostatectomy.

Co-variate	Hazard ratio	95% CI	P-value
Age	0.99	0.94–1.04	0.74
Gleason score ≤7	1 (ref)		
Gleason score ≥8	2.47	1.02–5.99	0.044
pT2a-pT2c	1 (ref)		
pT3a-pT3b	2.38	1.34–4.23	0.003
Surgical margin-negative	1 (ref)		
Surgical margin-positive	1.29	0.75–2.24	0.36
Positive cytoplasmic TLR9 expression	2.27	1.18–4.37	0.014

TLR9, Toll-like receptor-9; CI, confidence interval.

## References

[1] Jinushi M (2012). The role of innate immune signals in antitumor immunity. Oncoimmunology.

[2] Akira S, Hemmi H (2003). Recognition of pathogen-associated molecular patterns by TLR family. Immunol Lett.

[3] Hidmark A, von Saint Paul A, Dalpke AH (2012). Cutting edge: TLR13 Is a Receptor for Bacterial RNA. J Immunol.

[4] Chang YJ, Wu MS, Lin JT, Chen CC (2005). *Helicobacter pylori*-induced invasion and angiogenesis of gastric cells is mediated by cyclooxygenase-2 induction through TLR2/TLR9 and promoter regulation. J Immunol.

[5] Droemann D, Albrecht D, Gerdes J, Ulmer AJ, Branscheid D, Vollmer E, Dalhoff K, Zabel P, Goldmann T (2005). Human lung cancer cells express functionally active Toll-like receptor 9. Respir Res.

[6] Merrell MA, Ilvesaro JM, Lehtonen N, Sorsa T, Gehrs B, Rosenthal E, Chen D, Shackley B, Harris KW, Selander KS (2006). Toll-like receptor 9 agonists promote cellular invasion by increasing matrix metalloproteinase activity. Mol Cancer Res.

[7] Takala H, Kauppila JH, Soini Y, Selander KS, Vuopala KS, Lehenkari PP, Saarnio J, Karttunen TJ (2011). Toll-like receptor 9 is a novel biomarker for esophageal squamous cell dysplasia and squamous cell carcinoma progression. J Innate Immun.

[8] Kauppila JH, Takala H, Selander KS, Lehenkari PP, Saarnio J, Karttunen TJ (2011). Increased Toll-like receptor 9 expression indicates adverse prognosis in oesophageal adenocarcinoma. Histopathology.

[9] Väisänen MR, Väisänen T, Jukkola-Vuorinen A, Vuopala KS, Desmond R, Selander KS, Vaarala MH (2010). Expression of toll-like receptor-9 is increased in poorly differentiated prostate tumors. Prostate.

[10] Ronkainen H, Hirvikoski P, Kauppila S, Vuopala KS, Paavonen TK, Selander KS, Vaarala MH (2011). Absent Toll-like receptor-9 expression predicts poor prognosis in renal cell carcinoma. J Exp Clin Cancer Res.

[11] Jukkola-Vuorinen A, Rahko E, Vuopala KS, Desmond R, Lehenkari PP, Harris KW, Selander KS (2008). Toll-like receptor-9 expression is inversely correlated with estrogen receptor status in breast cancer. J Innate Immun.

[12] Ilvesaro JM, Merrell MA, Swain TM, Davidson J, Zayzafoon M, Harris KW, Selander KS (2007). Toll like receptor-9 agonists stimulate prostate cancer invasion in vitro. Prostate.

[13] Ren T, Xu L, Jiao S, Wang Y, Cai Y, Liang Y, Zhou Y, Zhou H, Wen Z (2009). TLR9 signaling promotes tumor progression of human lung cancer cell in vivo. Pathol Oncol Res.

[14] Sandholm J, Kauppila JH, Pressey C, Tuomela J, Jukkola-Vuorinen A, Vaarala M, Johnson MR, Harris KW, Selander KS (2012). Estrogen receptor-alpha and sex steroid hormones regulate Toll-like receptor-9 expression and invasive function in human breast cancer cells. Breast Cancer Res Treat.

[15] Kakonen SM, Selander KS, Chirgwin JM, Yin JJ, Burns S, Rankin WA, Grubbs BG, Dallas M, Cui Y, Guise TA (2002). Transforming growth factor-beta stimulates parathyroid hormone-related protein and osteolytic metastases via Smad and mitogen-activated protein kinase signaling pathways. J Biol Chem.

[16] Flatt T, Heyland A, Rus F, Porpiglia E, Sherlock C, Yamamoto R, Garbuzov A, Palli SR, Tatar M, Silverman N (2008). Hormonal regulation of the humoral innate immune response in *Drosophila melanogaster*. J Exp Biol.

[17] Tuomela J, Sandholm J, Karihtala P, Ilvesaro J, Vuopala KS, Kauppila JH, Kauppila S, Chen D, Pressey C, Harkonen P, Harris KW, Graves D, Auvinen PK, Soini Y, Jukkola-Vuorinen A, Selander KS (2012). Low TLR9 expression defines an aggressive subtype of triple-negative breast cancer. Breast Cancer Res Treat.

[18] Vincent IE, Zannetti C, Lucifora J, Norder H, Protzer U, Hainaut P, Zoulim F, Tommasino M, Trepo C, Hasan U, Chemin I (2011). Hepatitis B virus impairs TLR9 expression and function in plasmacytoid dendritic cells. PLoS One.

[19] Yu SL, Chan PK, Wong CK, Szeto CC, Ho SC, So K, Yu MM, Yim SF, Cheung TH, Wong MC, Cheung JL, Yeung AC, Li EK, Tam LS (2012). Antagonist-mediated down-regulation of toll-like receptors increases the prevalence of human papillomavirus infection in systemic lupus erythematosus. Arthritis Res Ther.

[20] González-Reyes S, Fernandez JM, González LO, Aguirre A, Suarez A, González JM, Escaff S, Vizoso FJ (2011). Study of TLR3, TLR4, and TLR9 in prostate carcinomas and their association with biochemical recurrence. Cancer Immunol Immunother.

[21] Ilvesaro JM, Merrell MA, Li L, Wakchoure S, Graves D, Brooks S, Rahko E, Jukkola-Vuorinen A, Vuopala KS, Harris KW, Selander KS (2008). Toll-like receptor 9 mediates CpG oligonucleotide-induced cellular invasion. Mol Cancer Res.

[22] Nakai Y, Nonomura N (2012). Inflammation and prostate carcinogenesis. Int J Urol.

[23] Kundu SD, Lee C, Billips BK, Habermacher GM, Zhang Q, Liu V, Wong LY, Klumpp DJ, Thumbikat P (2008). The toll-like receptor pathway: a novel mechanism of infection-induced carcinogenesis of prostate epithelial cells. Prostate.

[24] Di JM, Pang J, Sun QP, Zhang Y, Fang YQ, Liu XP, Zhou JH, Ruan XX, Gao X (2010). Toll-like receptor 9 agonists up-regulates the expression of cyclooxygenase-2 via activation of NF-kappaB in prostate cancer cells. Mol Biol Rep.

[25] Di JM, Pang J, Pu XY, Zhang Y, Liu XP, Fang YQ, Ruan XX, Gao X (2009). Toll-like receptor 9 agonists promote IL-8 and TGF-beta1 production via activation of nuclear factor kappaB in PC-3 cells. Cancer Genet Cytogenet.

